# Effect of Repetitive Bending and Straightening Process on Microstructure Properties and Deformation Mechanism of a Ti–Al–Cr–Mo Alloy

**DOI:** 10.3390/ma16216873

**Published:** 2023-10-26

**Authors:** Zhuoliang Li, Yan Xu, Jiang Qian, Linhong Song

**Affiliations:** HuiBo Heat Energy Engineering Technology Center, Shenyang Academy of Instrumentation Science Co., Ltd., Shenyang 110043, China; 18842621065@163.com (Y.X.); qianjiang127@163.com (J.Q.);

**Keywords:** titanium alloy, repetitive bending and straightening, mechanical properties, grain refinement, dislocation behavior

## Abstract

In this research, a repetitive bending and straightening process was carried out on the Ti–3Al–4Cr–Mo alloy for 20 passes. The changes in mechanical properties of the titanium alloy before and after repetitive bending and annealing were studied. The microstructure evolution and deformation mechanism were analyzed. The results show that after the repetitive bending and straightening process, the microstructure of the Ti–3Al–4Cr–Mo alloy is obviously refined, and, simultaneously, the yield strength is significantly improved. After annealing at 850 °C, the plastic ductility of the material was improved. The combined effects of grain refinement and dislocation behavior were the main reasons for the improvement in mechanical properties of the Ti–3Al–4Cr–Mo alloy. Twinning rarely occurred during plastic deformation of the Ti–3Al–4Cr–Mo alloy. The fine grains strongly inhibited the formation of twins. In addition, a small amount of α to β phase transformation was observed during the repetitive bending and straightening process of the material, which may have been induced by strain accumulation.

## 1. Introduction

High strength, low density, excellent corrosion resistance, and good high-temperature creep resistance are the main characteristics of titanium alloys. These characteristics make titanium and its alloys attractive in the aerospace, chemical, medical, and automotive fields, among many others [1,2,3]. The Ti–3Al–4Cr–Mo alloy is a near-α-type titanium alloy with medium strength at a low temperature. This kind of alloy has excellent weldability and cold/hot formability in the annealed state, and relatively high strength plastic volume is maintained at 20 K temperature. It is an ideal material for key components of aerospace engines. However, as a typical α-type titanium alloy, the Ti–3Al–4Cr–Mo alloy has poor plastic deformation ability at room temperature (elongation is less than 10%), and it is difficult to form relatively complex parts. This seriously limits the industrial application of this material in the aerospace field.

As a severe plastic deformation method suitable for sheet and strip, the repetitive corrugation and straightening (RCS) process can introduce severe plastic deformation without changing the original size of the sheet (especially the thickness of the sheet). Thus, the microstructure of the material can be significantly refined and the mechanical properties can be improved. Research [4,5] showed that the RCS process had a significant effect on the microstructure refinement of the surface layer of the sheet, and it could also effectively improve the comprehensive mechanical properties of the material. Huang et al. [6] prepared nanocrystalline copper by the RCS process and studied the dislocation behavior during the deformation process. Some unique microstructural features such as isolated dislocation cells, dislocation tangle zones, and uncondensed dislocation walls were revealed. Rajinikanth et al. [7] studied the strength changes of Al and Al–0.2Sc materials during RCS, and the mechanisms of the influence of different process parameters on the strength of Al and Al alloys were quantitatively analyzed. Thangapandian et al. [8] studied the influence of RCS on grain size refinement and deformation mechanism of the AA6063 aluminum alloy under different temperature conditions. The results showed that the room temperature tensile strength and hardness values of the processed material decreased with the increasing processing temperature, and the transformation of low-angle grain boundaries (LAGBs) to high-angle grain boundaries (HAGBs) and dislocation tangles were highly dependent on the strain imparted, which could be controlled by selecting the proper processing temperature. Up to now, research on the RCS process has been relatively uncommon, and most of the research focused on FCC and BCC metals such as Cu alloys and Al alloys. In addition, the RCS process accumulates strain by bending the sheet into a “V” shape or close to a “V” shape, resulting in the heterogeneous accumulation of strain. Only local positions of the sheet can be strengthened, which results in the impossibility of universal industrial applications of the RCS process.

In the present study, the RCS process was improved. Continuous bending deformation was used instead of “corrugation” to achieve a uniform distribution of strain in the length direction of the sheet. In this study, a Ti–3Al–4Cr–Mo alloy was put through the RBS (repetitive bending and straightening) process for multiple passes at room temperature. The mechanical properties and microstructure evolution of the material before and after deformation and heat treatment were studied. The deformation and strengthening mechanism of the Ti–3Al–4Cr–Mo alloy during the RBS process was revealed.

## 2. Experimental Procedure

**Figure 1:**
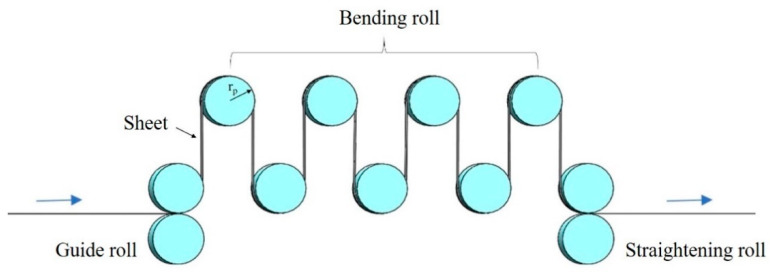
Schematic of repeated bending and straightening process.

**Figure 2:**
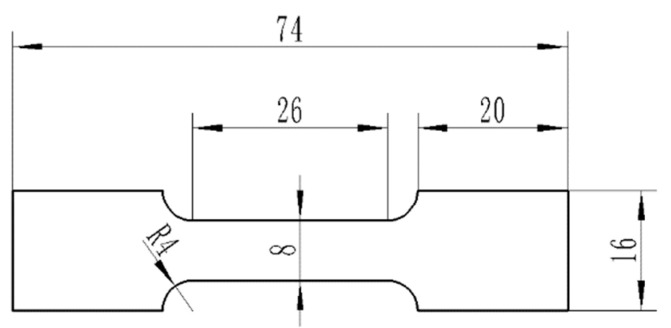
Schematic of the tensile samples.

In the present study, a Ti–3Al–4Cr–Mo rolled sheet with a thickness of 0.3 mm and width of 300 mm was used and the continuous RBS process was conducted along the rolling direction. The chemical component is shown in Table 1. As shown in Figure 1, the forward and backward bending of the sheet was defined as one pass, and the sheet was straightened after 20 passes of deformation. The bending radius (r_p_) of RBS was 25 mm. The Ti–3Al–4Cr–Mo sheet that had gone through the RBS process was annealed to obtain better forming properties. The annealing temperature was 850 °C and the sheet was held for 1 h at this temperature condition. Mechanical properties were tested and the microstructures were characterized on the original sheet, the sheet that had gone through the RBS process, and the annealed sheet. The tensile samples were prepared along the rolling direction by wire cutting; the sizes of the tensile sample are shown in Figure 2. The tensile tests were carried out on the SANSCMT5000 electronic universal material test machine at the same elongation speed, at room temperature (293 K) with the initial strain rate of 1.0 × 10^−3^ s^−1^. More than three tensile samples were tested under each condition to ensure the reliability of the test results. Vickers microhardness tests were performed on the polished surface, which was polished using abrasive papers and metallographic grinding paste. The test load was 10 gf, and the loading time was 10 s. The distance between two adjacent test points was 1 mm. The average of 10 independent test results was taken as the microhardness value of the sample. After being ground using abrasive papers, the samples were polished using argon ion at −30 °C. Then, the electron backscatter diffraction (EBSD) characterizations were tested using ultra plus field emission scanning electron microscope. The XRD tests were carried out on the TD-3500 X-ray diffractometer with the scanning angle of 30°~90° and the scanning rate of 1°/min.

## 3. Experimental Results

During the repetitive bending and straightening processes, the deformation form of the material was approximately simple bending. The strain accumulation gradually decreased from surface to neutral layer. According to the research of Liu et al. [9], the tangential strain of any small unit of the sheet during bending can be expressed as follows:

(1) $$\varepsilon_{P}=ln\frac{r_{P}}{r_{0}}$$

and the equivalent strain is:

(2) $$\varepsilon^{T}=\frac{2}{\sqrt{3}}\left|\varepsilon_{P}\right|$$

where $r_{P}$ is the radius of curvature, and $r_{0}$ is the distance from the center of curvature to the neutral layer of the sheet.

It could be obtained by calculation that after experiencing the RBS process 20 times, obvious plastic deformation occurred on the surface of the sheet, and the cumulative strain reached ~0.35. A large number of studies [10,11,12] have shown that with the continuous accumulation of strain, the microstructure of titanium alloys can be significantly refined, and the ductility and yield strength of the material can be improved. In this study, the Ti–3Al–4Cr–Mo sheet was bent, and the cumulative strain was mainly concentrated on the surface of the material, which had a significant effect on the microstructure and mechanical properties of the surface of the material, but a relatively weak effect on the internal microstructure near the neutral layer, forming a gradient material with gradually changing properties from the surface to the central layer. This study focuses on the effects of the RBS process on the microstructure and mechanical properties of the Ti–3Al–4Cr–Mo sheet.

**Figure 3:**
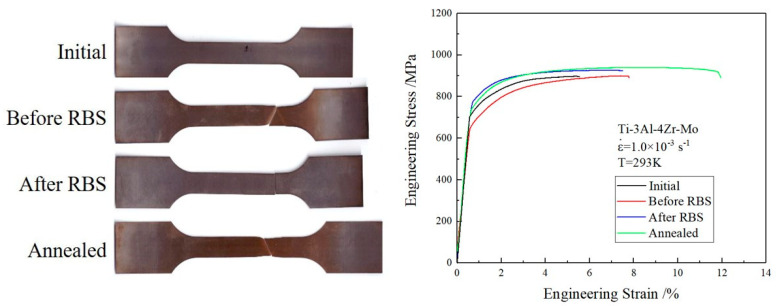
Tensile specimens and engineering strain–engineering stress curves of Ti–3Al–4Cr–Mo alloy under different conditions.

As shown in Figure 3, the mechanical properties of the Ti–3Al–4Cr–Mo alloy changed obviously after RBS and annealing. After 20 passes through the RBS process, the yield strength of the material increased from 650 MPa to 772 MPa, while the elongation did not decrease significantly (~7.5%). In order to further improve the comprehensive mechanical properties of the Ti–3Al–4Cr–Mo material, annealing was carried out at 850 °C on the material after RBS for 1 h and high purity argon gas (>99.999%) was used to protect the material from oxidation. It can be seen from the engineering strain–engineering stress curves (Figure 3) that the deformation ability of the material is significantly enhanced after annealing, and the elongation reaches ~12%. At the same time, the yield strength of the material was slightly reduced (748 MPa), which was still significantly higher than that of the initial material. In addition, as shown in Table 2, the Vickers microhardness of the Ti–3Al–4Cr–Mo sheet before RBS was only 287.7 Hv. After 20 passes through the RBS process, the microhardness of the material was significantly increased, reaching 423.6 Hv. After annealing, the microhardness of Ti–3Al–4Cr–Mo decreased (326.6 Hv), but it was still significantly higher than that of the initial material. Therefore, after RBS and heat treatment, the microhardness, yield strength and ductility of Ti–3Al–4Cr–Mo were significantly increased. The comprehensive mechanical property of the material was significantly improved.

**Figure 4:**
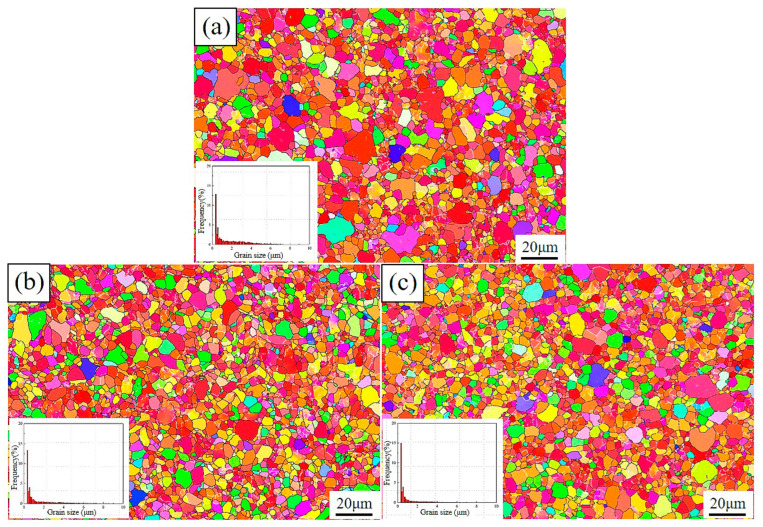
Microstructures of Ti–3Al–4Cr–Mo alloy under different conditions. (**a**) Before RBS, (**b**) after RBS, (**c**) annealed.

**Figure 5:**
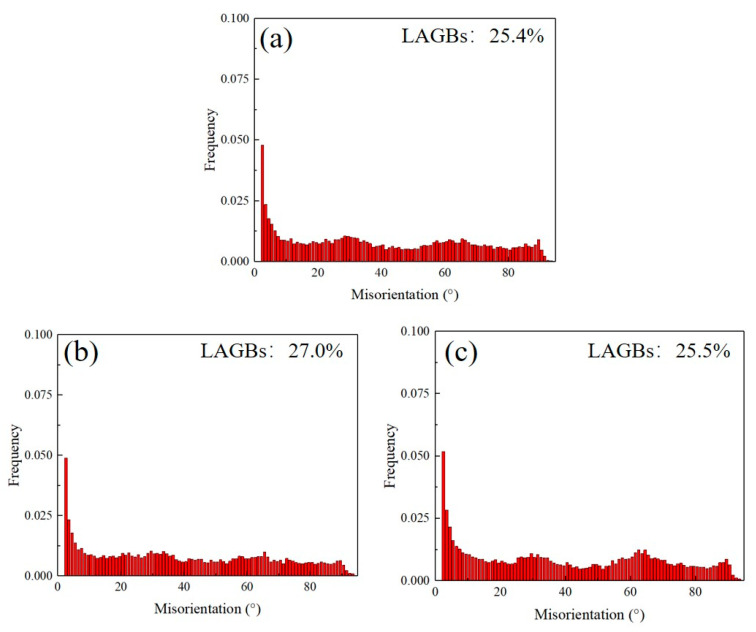
Misorientation distribution of Ti–3Al–4Cr–Mo alloy under different conditions. (**a**) Before RBS, (**b**) after RBS, (**c**) annealed.

Figure 4 shows the microstructure and grain size distribution of the Ti–3Al–4Cr–Mo material under different conditions. It can be seen from the figure that the initial grains of the material were relatively fine, but not uniform. Fine (grain size < 4 μm) and coarse grains existed in the material at the same time. The average grain size of the initial Ti–3Al–4Cr–Mo material was about 4.5 μm. After 20 times of experiencing bending and straightening, the surface microstructure of the material was obviously refined. The coarse grains were gradually broken into fine grains during plastic deformation, and the average grain size of the material was reduced to ~3.7 μm. Compared with the initial material, the microstructure after plastic deformation was obviously more uniform. After further heat treatment, the microstructure of the Ti–3Al–4Cr–Mo alloy did not change significantly. Only some grains grew, and the average grain size of the material was ~3.8 μm. Using the EBSD method, the crystal orientation of each grain or sub-grain in the region being examined could be obtained. Thus, the misorientation data between adjacent grains or sub-grains (grain boundary or sub-boundary) could be obtained, too. The misorientation distribution difference of the material can be obtained through the statistics of the detection data in several regions. It can be seen from the misorientation distribution diagrams of the alloys under different conditions (Figure 5) that the low-angle grain boundary contents of the initial material, the deformed material, and the heat-treated material are 25.4%, 27.0%, and 25.5%, respectively.

**Figure 6:**
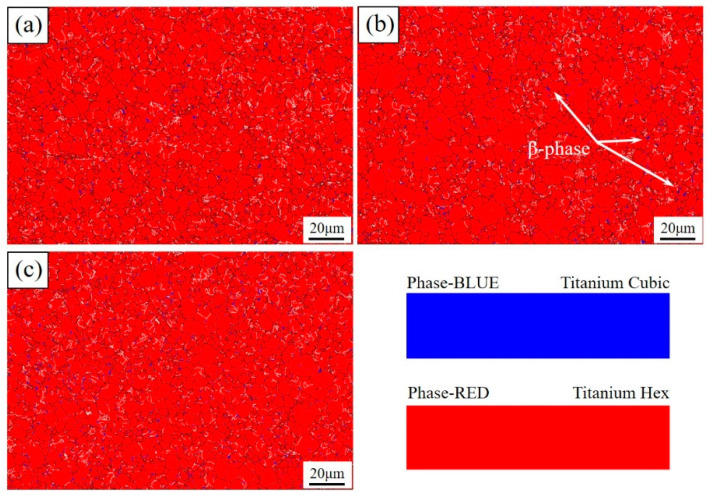
Phase distribution diagrams of Ti–3Al–4Cr–Mo alloy under different conditions. (**a**) Before RBS, (**b**) after RBS, (**c**) annealed.

The information of the phase distribution of the material could be obtained by EBSD. As shown in Figure 6, as a typical near-alpha-type titanium alloy, the phase composition of the Ti–3Al–4Cr–Mo material was mainly α-Ti under different conditions. The β phase content of the Ti–3Al–4Cr–Mo alloy was only 1.02% in the initial alloy, and it reached 1.56% and 1.72%, respectively, after repeated bending and heat treatment. The plastic deformation and heat treatment both led to an increase in β phase content.

**Figure 7:**
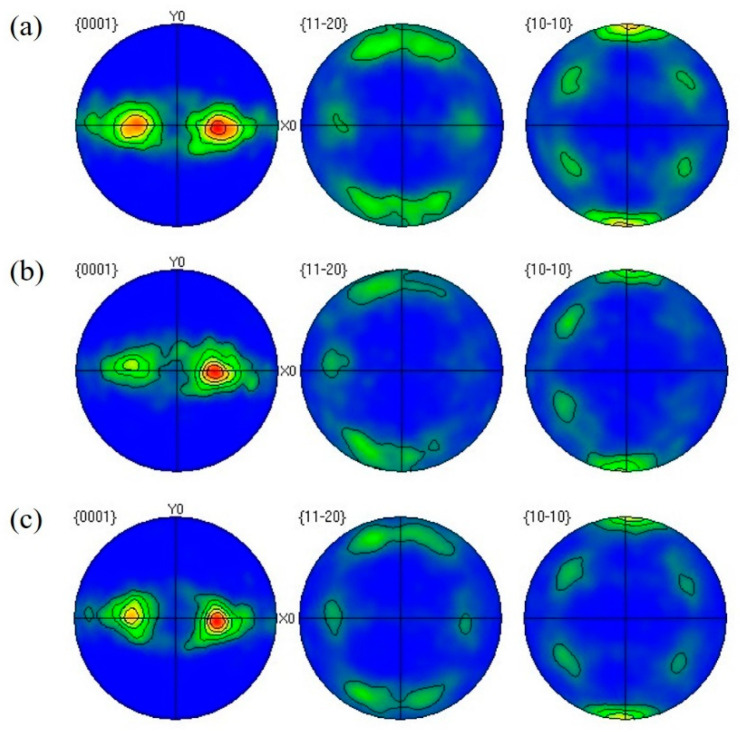
Pole figures of Ti–3Al–4Cr–Mo alloy under different conditions. (**a**) Before RBS, (**b**) after RBS, (**c**) annealed.

Figure 7 shows the pole diagrams of the Ti–3Al–4Cr–Mo alloy under different conditions. The initial material had a strong $\left(10\bar{1}1\right)$ slant texture due to the preparation rolling process, with a maximum texture intensity of 9.1. After the RBS process and heat treatment, the texture type of the titanium alloy had not changed significantly, but the texture intensity had increased. After plastic deformation and heat treatment, the maximum values of texture intensities of the material reached 12.0 and 12.3, respectively. It could be seen that the plastic deformation process led to an increase in texture intensities. Although grain growth occurred during heat treatment, the texture intensity did not change significantly.

**Figure 8:**
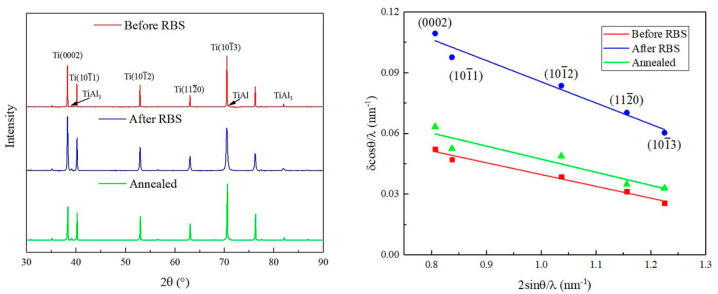
XRD patterns and 2 sin θ/λ-δ cos θ/λ curves of Ti–3Al–4Cr–Mo alloy under different conditions.

Figure 8 shows the XRD results of the Ti–3Al–4Cr–Mo alloy under different conditions. According to Dragomir’s theory [13], the dislocation density inside the material can be obtained by XRD. As shown in Figure 8, the width of the diffraction peak is affected by the grain size, while the dislocation multiplication is caused by the micro-strain inside the material [14]. The micro-strain can be obtained from the slope of the 2 sin θ/λ-δ cos θ/λ curve. Therefore, the dislocation density of the sample can be obtained according to Formula (3) [13].

(3) $$\rho ≅\frac{4\pi e^{2}}{Cb^{2}}$$

where *e* is the micro-strain, *b* is the Burgers vector of pure Ti, and *C* is the coefficient. In order to improve the calculation accuracy, the full-width at the half of the maximum (FWHM) of the XRD equipment was corrected, and the correction curve of the instrument was obtained. By subtracting the instrument FWHM from the XRD measurement results of titanium alloy, the true FWHM, δ, after the widening effect caused by micro-strain, could be obtained. XRD data of multiple samples under the same parameters were processed in the same process, and the obtained results were averaged to further improve the reliability of the calculated results.

The dislocation densities of the initial materials, deformed materials, and heat-treated materials were 0.56 × 10^12^ cm^−2^, 1.82 × 10^12^ cm^−2^, and 0.69 × 10^12^ cm^−2^, respectively. It can be seen that after plastic deformation, the dislocation density inside the material is significantly increased. After annealing, the density of the dislocation decreased rapidly, but its value was still slightly higher than that of the original material.

## 4. Discussion

The microstructure and mechanical properties of the studied Ti–3Al–4Cr–Mo alloy significantly changed after RBS and heat treatment. The surface grains of the material were broken by repetitive bending deformation, and the average grain size was refined from 4.5 μm to 3.7 μm. As a typical α-type titanium alloy, the main mechanism of plastic deformation of the Ti–3Al–4Cr–Mo alloy was basal slip at room temperature. Dislocations multiplied and intertwined with each other during deformation processes, and dislocation walls were gradually formed inside the grain. The dislocations on both sides of the dislocation wall moved through the grain interior and were absorbed by the dislocation walls. This process was repeated over and over again, resulting in a gradual disorientation difference on both sides of the dislocation walls, forming low-angle grain boundaries. The low-angle grain boundaries formed a network of “frames” inside the grain, which divided the grains into many cellular blocks. With the increase in strain accumulation, dislocations were continuously absorbed by low-angle grain boundaries, which gradually transformed low-angle grain boundaries into high-angle grain boundaries, and grains were gradually refined due to the formation of high-angle grain boundaries [15,16]. According to the Hall–Petch relationship, the yield strength of the alloy is inversely proportional to the square root of its grain size. With the decrease in grain size, the yield strength of the titanium alloy was significantly improved, and, simultaneously, the plasticity of the material increased [17]. On the other hand, the plastic deformation process led to dislocation multiplication. The contribution of dislocation density to the strength could be expressed by Taylor’s relationship (Equation (4)) [18]:

(4) $$\sigma_{dis}=M\alpha Gb\sqrt{\rho }$$

where *M* is the Taylor factor of titanium alloy; *α* is a dimensionless constant with order 1, which represents the interaction between the basal plane dislocations. *G* is the shear modulus; *b* is the Burgers vector of perfect base plane dislocations; *ρ* is the dislocation density of the material. In the present study, grain refinement, an increase in the low-angle grain boundaries, and the enhancement of the dislocation density were all observed during the RBS process. These indicate that both grain refinement and dislocation multiplication make contributions to the yield strength. On the other hand, grain refinement also results from dislocation behavior. As mentioned previously, the formation of low-angle grain boundaries was due to the dislocation multiplication caused by deformation, and the uniform “frames” formed inside the grains ensured that the Ti–3Al–4Cr–Mo material after experiencing the RBS process maintained a fine, uniform, and equiaxial microstructure. It can be seen that the increase in dislocation density can improve the yield strength of the titanium alloy to a certain extent, but the effect is not significant. Grain refinement was the main factor affecting the yield strength of materials in the present study. It could be inferred that with the continuation of the RBS process, strain would accumulate continuously inside the material, further grain refinement and dislocation multiplication could occur, and the yield strength of the material would be further improved. After heat treatment, some grains became bigger, the grain boundary strengthening effect weakened, and the yield strength of the material decreased slightly.

Many studies have shown that grain refinement can effectively improve the deformation coordination ability of the metal with a HCP structure, reduce the tendency of stress concentration, and inhibit the initiation and extension of cracks. Thus, the plastic deformation ability of materials can be improved [19,20,21]. As a typical α-Ti alloy, the dominate lattice type of the Ti–3Al–4Cr–Mo material was a HCP structure, and basal slip was the most important plastic deformation mechanism. The plastic deformation ability of this alloy was poor at room temperature (elongation at break ~5%). Although grain refinement of the surface layer improved the deformation coordination and crack inhibition ability of the material, the increase in dislocation density caused by plastic deformation reduced the inner space of the grain and inhibited the slip process of dislocations. Therefore, the elongation at the break of the sample after repetitive bending deformation was slightly lower than that of the initial sample. Under the combined effect of grain refinement and the decrease in dislocation multiplication potential, the elongation at the break of the material after RBS basically remained unchanged compared to the initial sample. After heat treatment, the dislocation density of the material decreased rapidly, and there was enough space in the grains for the slip of the dislocations. The deformation ability of the Ti–3Al–4Cr–Mo material was significantly improved under the comprehensive effect of dislocation slip and grain refinement.

At room temperature, the deformation mechanism of titanium alloys mainly includes basal slip and twinning [22,23,24]. In this study, the Ti–3Al–4Cr–Mo alloy did not exhibit obvious twinning behavior during deformation and heat treatment (Figure 5). This was mainly related to the dislocation multiplication space after grain refinement. Li’s study shows that the formation of twins in HCP metals requires the material to have a certain grain size, and only when the grain size is larger than the critical grain size can the twins stably nucleate and grow [25]. Figueiredo et al. [26] and Jimenez et al. [27] both proved that there was a transition from twinning to slip of the dominated mechanism with the decreasing grain size. According to the theoretical model of Yu et al. [28], the dislocations that were perpendicular to the slip plane of the twins would play the role of ‘promoters’ to activate the growth of the twins. These ‘promoters’ stimulated the twinning slip planes layer-by-layer, leading to the growth of the twins step-by-step at the length of one Burgers vector at a time towards the slip direction. For magnesium alloys, the critical grain size for stable growth of twins is about 5.9 μm [29]. Similar to magnesium alloys, titanium alloys are also HCP structures, which means Yu’s theory also applies here. However, until now, no work in the literature has reported the twinning critical grain size of titanium alloys. In this study, the average grain size of Ti–3Al–4Cr–Mo in different states was less than 4.5 μm. It can be seen from Figure 4 and Figure 6 that large amounts of fine grains with a size below 4 μm exist in the material. The high grain boundary content caused easy contact of dislocations to the grain boundaries. The dislocations would be released easily during the slip process. It can be inferred that small grains could not provide enough space for stable nucleation and growth of the twins, so the twinning process was strongly inhibited.

As can be seen from Figure 6, after repetitive experience of the bending and deformation process, the accumulative strain of the Ti–3Al–4Cr–Mo sheet reached ~0.35, and the content of β phase also increased. In the region with fine grains and high content of low-angle grain boundaries especially (Figure 6b), the content of the β phase was significantly higher than that of undeformed samples. These regions generally had large strain, high local energy, and residual stress. It could be inferred that the strain-induced phase transformation process occurred inside the material. At present, there is no work in the literature reporting on this process, and an accurate mechanism description is also needed through further studies. In the present study, the process of strain-induced phase transition was not significant, which may be due to the relatively low accumulation of strain accumulation. With the increase in strain accumulation, a more obvious strain-induced phase transition may occur.

In addition, the pole diagrams of the Ti–3Al–4Cr–Mo material under different conditions (Figure 7) show that no obvious grain rotation occurs during plastic deformation. The increase in texture intensity was mainly due to the grain refinement and the increasing grain boundary content. The $\left(10\bar{1}1\right)$ slope texture caused an angle of ~56° between slipping and deformation direction, which had a certain softening effect on the material and was conducive to the plastic deformation [30]. However, the evolution of texture only occurred in the surface layer of the material, and the change in texture intensity was not significant, so the influence on the mechanical properties of the material was weak.

## 5. Conclusions

1. After 20 passes through the RBS process, the microstructure of Ti–3Al–4Cr–Mo was uniform and fine, and the yield strength was obviously improved. The average grain size of the surface layer was reduced from 4.5 μm to 3.7 μm. After annealing, the elongation of the material increased significantly and the yield strength decreased, but it was still significantly higher than that of the initial material;
2. With the accumulation of equivalent strain, the dislocation density of the Ti–3Al–4Cr–Mo alloy was increased. The moving dislocation formed a low-angle grain boundary “frame” inside the grain. Low-angle grain boundaries absorbed dislocations to form high-angle grain boundaries, which led to grain refinement. The effect of grain refinement and dislocation behavior led to an increase in the mechanical properties of the materials;
3. The deformation process of the studied titanium alloy did not involve twinning, mainly because the fine grains could not provide enough space for the stable nucleation and growth of twins. The twinning process was strongly inhibited;
4. After experiencing RBS processes, the strain accumulated continuously in the surface layer of the Ti–3Al–4Cr–Mo alloy, and the content of the β phase increased. The process of strain-induced phase transition may have occurred inside the material.

## Figures and Tables

**Table 1:** Chemical composition of the Ti–3Al–4Cr–Mo alloy.

Element	Al	Cr	Mo	Fe	Ti
wt%	3.4	4.1	0.9	0.12	Balance

**Table 2:** Vickers microhardness of Ti–3Al–4Cr–Mo sheet under different conditions.

	Before RBS	After RBS	Annealed
Microhardness (Hv)	287.7	423.6	326.6

## Data Availability

Not applicable.
